# Überproportional angestiegene Inzidenz proximaler Femurfrakturen in einem Level-One-Traumazentrum: Epidemiologische Analyse von 2016 bis 2022

**DOI:** 10.1007/s00113-023-01359-0

**Published:** 2023-08-29

**Authors:** Philipp Schippers, Erol Gercek, Felix Wunderlich, Jochen Wollstädter, Yama Afghanyar, Charlotte Arand, Philipp Drees, Lukas Eckhard

**Affiliations:** Zentrum f. Orthopädie und Unfallchirurgie, Universitätsmedizin Mainz, Langenbeckstr. 1, 55131 Mainz, Deutschland

**Keywords:** Unfallchirurgie, Proximale Femurfrakturen, Patientenaufkommen, Versorgungsstruktur, Arbeitspensum, Trauma surgery, Proximal femoral fractures, Patient volume, Supply structure, Workload

## Abstract

**Hintergrund:**

Proximale Femurfrakturen stellen mit mehr als 20 % die häufigste Frakturentität in Deutschland dar. Gleichzeitig müssen proximale Femurfrakturen aufgrund eines Beschlusses des Gemeinsamen Bundesausschusses (G-BA) von 2019 innerhalb von 24 h operiert werden. Um einen subjektiv wahrgenommen Anstieg des Arbeitspensums in der Unfallchirurgie an einem überregionalen Traumazentrum (ÜTZ) zu quantifizieren, wurde die Anzahl der proximalen Femurfrakturen von 2016 bis 2022 analysiert. Proximale Femurfrakturen wurden hierfür aufgrund ihrer Häufigkeit und der Homogenität in der Behandlung ausgewählt.

**Methode:**

Anhand der ICD-10-Diagnosen wurden alle operierten proximalen Femurfrakturen der Jahre 2016–2022 mitsamt der Postleitzahl an einem ÜTZ ausgewertet.

**Ergebnis:**

Die Anzahl der operativ versorgten proximalen Femurfrakturen ist von 2016 bis 2022 um 100 % gestiegen. Der größte Anstieg wurde mit 60 % von 2020 bis 2022 verzeichnet. Gleichzeitig kam es zu einer deutlichen Vergrößerung des Einzugsradius der versorgten Patienten.

**Schlussfolgerung:**

Am untersuchten ÜTZ kam es im (inter-)nationalen Vergleich zu einem überproportionalen Anstieg der versorgten proximalen Femurfrakturen. Der Anstieg des Einzugsradius und die Zunahme der versorgten Patienten im Stadtgebiet zeigen, dass immer weniger Krankenhäuser an der Notfallversorgung teilnehmen. Mögliche Erklärungen sind ein Ressourcenmangel, verstärkt durch die COVID-19-Pandemie und den Fachkräftemangel, Schnittstellenproblematiken an Bundesländergrenzen oder strenge Vorgaben des G‑BA in der Versorgung der proximalen Femurfrakturen. Es ist bei gleich gebliebener Infrastruktur im untersuchten ÜTZ von einem deutlich erhöhten Arbeitsaufkommen für alle beteiligten Professionen auszugehen.

**Graphic abstract:**

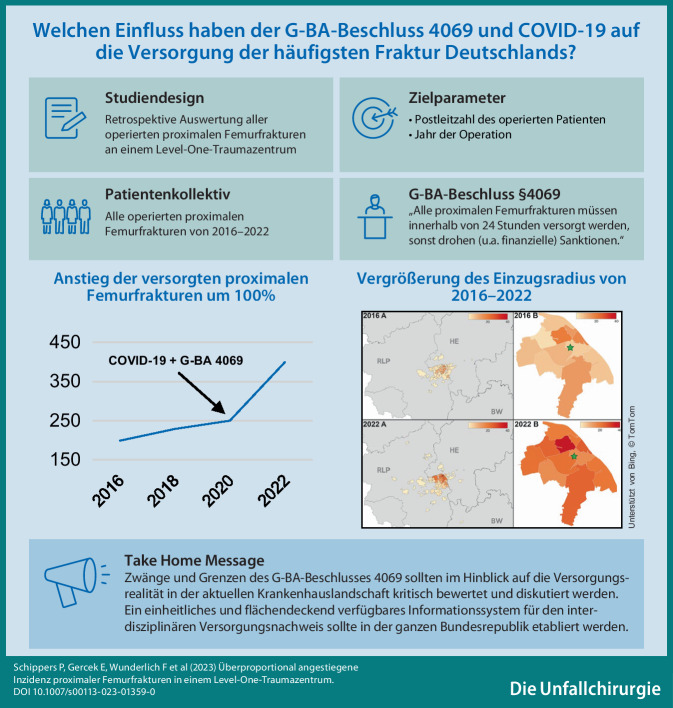

## Abstract (en)

**Background:**

Proximal femoral fractures represent the most frequent fracture entity in Germany accounting for more than 20% of all fractures. According to a decision of the Federal Joint Committee from 2019, proximal femoral fractures also have to be surgically treated within 24 h. In order to quantify a subjectively perceived increase in workload in trauma surgery at a supraregional trauma center, a retrospective analysis of the number of surgically treated proximal femoral fractures was performed. Proximal femoral fractures were chosen due to their high incidence and homogeneous treatment.

**Methods:**

Using ICD-10 codes, all surgically treated proximal femoral fractures from 2016 to 2022, including the patient’s zip code, were retrieved from the database of the trauma center.

**Results:**

The number of surgically treated proximal femoral fractures doubled from 2016 to 2022. The highest increase (60%) was recorded from 2020 to 2022. Heat maps show an increase in the catchment area radius as well.

**Conclusion:**

When compared (inter)nationally, a disproportionate increase in the amount of surgically treated proximal femoral fractures was recorded at the trauma center studied. The increase of the catchment area radius and the number of patients treated in the urban area show that less and less hospitals participate in emergency treatment. Possible explanations are a lack of resources aggravated by the recent COVID-19 pandemic and a lack of qualified personnel, interface problems between the federal states or the strict requirements of the Federal Joint Committee in the treatment of proximal femoral fractures. It must be assumed that there is a clearly increased workload for all professions involved in the trauma center investigated, although the infrastructure has remained unchanged.

Proximale Femurfrakturen sind die häufigste Frakturentität und betreffen v. a. ältere Patienten. In Anbetracht der hohen Morbidität bei verzögerter Versorgung müssen proximale Femurfrakturen seit einem Beschluss des Gemeinsamen Bundesausschusses (G-BA) innerhalb von 24 h operativ versorgt sein. Aufgrund der hohen Inzidenz und der homogenen Versorgung wurden proximale Femurfrakturen in dieser Studie verwendet, um die Veränderung des Arbeitspensums an einem überregionalen Traumazentrum in den letzten 6 Jahren abzubilden.

## Einleitung

Proximale Femurfrakturen stellen die häufigste Frakturentität bei Erwachsenen dar. Die Inzidenz der Schenkelhals- und der pertrochantären Frakturen lag 2019 in Deutschland bei über 150.000. Gemeinsam machten sie damit 22,6 % aller Frakturen aus [8].

Proximale Femurfrakturen stellen in vielerlei Hinsicht eine Herausforderung in der Versorgung dar. In der überwiegenden Mehrzahl der Fälle ist eine operative Therapie zur Wiederherstellung der Mobilität oder zumindest der schmerzarmen pflegerischen Versorgung der zumeist geriatrischen Patienten indiziert. Aufgrund der steigenden Mortalität bei zunehmender Verzögerung der operativen Versorgung hat diese gemäß dem Beschluss 4069 des G‑BA vom 22.11.2019 innerhalb von 24 h zu erfolgen. Kann die versorgende Klinik diese Vorgabe nicht einhalten, drohen finanzielle Sanktionen bis hin zum Entzug der Versorgungserlaubnis [4]. Hierdurch entsteht aufgrund der zeitlichen Unvorhersehbarkeit proximaler Femurfrakturen eine logistische Herausforderung für die an der unfallchirurgischen Notfallversorgung teilnehmenden Kliniken. Elektiv geplante Eingriffe müssen teils aufgrund der ausstehenden Versorgung einer proximalen Femurfraktur verschoben oder gänzlich abgesagt werden. Da v. a. geriatrische Patienten betroffen sind, ist die Behandlung oftmals durch Komorbiditäten und die Einnahme von Antikoagulanzien resp. Thrombozytenaggregationshemmern verkompliziert [13]. Dies führt weiterhin zu steigenden Behandlungskosten mit verlängerter Liegedauer [5], was aktuelle Problematiken wie den Bettenmangel aufgrund des Pflegenotstands [6] verschärft. Proximale Femurfrakturen stellen somit eine besondere Frakturentität mit sehr hohem Bereitstellungs- und Versorgungsaufwand dar.

Aufgrund des hohen Versorgungsaufwandes und der Homogenität des gesetzlich vorgegebenen Zeitfensters bei operativer Behandlung eignen sich proximale Femurfrakturen als Indikator für die Belastung eines Krankenhauses bzw. einer unfallchirurgischen Abteilung und deren Mitarbeitern. Hypothese der Autoren ist, dass, verstärkt durch die COVID-19-Pandemie sowie Betten- und Personalmangel, immer weniger „kleinere“ Krankenhäuser an der Versorgung unfallchirurgischer Patienten mit proximalen Femurfrakturen teilnehmen. Hierdurch kommt es zu einem drastischen Anstieg des Patientenaufkommens von überregionalen Traumazentren (ÜTZ), welche sich der Notfallversorgung, auch im Falle bereits ausgereizter Kapazitäten, nicht entziehen können. Dies führt unausweichlich zu einer höheren interprofessionellen Arbeitsbelastung und zu der Problematik, dass die vom G‑BA gesetzten Zeitfenster immer schwieriger einzuhalten sind.

## Methodik

Es wurde eine Datenabfrage mit dem Krankenhausinformationssystem (KIS) durchgeführt. Hierbei wurden die Postleitzahlen der operierten Patienten der Jahre 2016–2022 ausgegeben, bei denen eine ICD-10-Diagnose von S72.00–S72.11 verschlüsselt wurde. Durch die ICD-10-Diagnosen S72.0x sind Schenkelhalsfrakturen abgedeckt, mit S72.1x pertrochantäre Frakturen. Im nächsten Schritt wurde jeweils eine Pivot-Tabelle aus den Rohdaten der einzelnen Jahre erzeugt. Hierdurch konnte die Anzahl der versorgten Frakturen pro Postleitzahl angezeigt werden. Für die grafische Aufarbeitung wurde das Kartogramm von Excel® (Microsoft®, Seattle, USA) mit der Unterstützung von Bing® (Microsoft®, Seattle, USA) und TomTom® (TomTom N.V., Amsterdam, Niederlande) verwendet. Die Definition des Stadtgebietes bezieht sich auf eine Summe aus 11 Postleitzahlen, welche offiziell zu dem Stadtgebiet zählen, in dem sich auch die untersuchte Klinik befindet.

Mithilfe einer weiteren Datenabfrage des KIS wurde die Anzahl der als „notfallmäßige Vorstellung“ gebuchten Konsultationen in der Notaufnahme der untersuchten Klinik der Jahre 2016–2022 ausgelesen. Hierbei wurden, aufgrund der Inhomogenität der Verbuchungen und der Veränderung dieser im Laufe der Jahre, andere Konsultationen wie Wiedervorstellungen, Kindernotfälle, Arbeitsunfälle und konsiliarische Vorstellungen nicht einberechnet.

Die untersuchte Klinik ist als ÜTZ Mitglied in einem *TraumaNetzwerk* der DGU®. TraumaNetzwerke sind Zusammenschlüsse von Kliniken unterschiedlicher Versorgungsstärke, die sich in geografischer Nähe zueinander befinden. Ziel ist vorrangig, die Sicherstellung der flächendeckenden Versorgung von Schwerstverletzten zu gewährleisten. Weiterhin ermöglicht das TraumaNetzwerk eine enge Zusammenarbeit der teilnehmenden Kliniken, u. a. in den Bereichen der Aus‑, Fort- und Weiterbildung sowie der Telemedizin. Im Bundesland der untersuchten Klinik gibt es 6 TraumaNetzwerke mit teilweise großen Überschneidungen der Versorgungsgebiete. Im TraumaNetzwerk der untersuchten Klinik gibt es 6 zertifizierte Kliniken: Die untersuchte Klinik als überregionales Traumazentrum, 2 regionale Traumazentren und 3 lokale Traumazentren [3]. In unmittelbarer Nachbarschaft der untersuchten Klinik befindet sich ein TraumaNetzwerk mit 6 überregionalen Traumazentren, wobei eines hiervon weniger als 10 km (Luftlinie) entfernt ist. Im benachbarten TraumaNetzwerk wird im gesamten Bundesland eine Informationstechnologie zum interdisziplinären Versorgungsnachweis (IVENA eHealth, mainis IT-Service GmbH, Offenbach am Main) verwendet. Dieselbe Technologie wird mittlerweile in 9 weiteren Bundesländern eingesetzt, wobei in Grenzgebieten der teilnehmenden Bundesländer ein grenzübergreifender Informationsaustausch möglich ist. Im TraumaNetzwerk der untersuchten Klinik hingegen wird ein im Bundesland entwickeltes System mit landesweitem Behandlungskapazitätennachweis geführt, ohne Austausch zu den benachbarten Bundesländern.

## Ergebnisse

**Figure 1:**
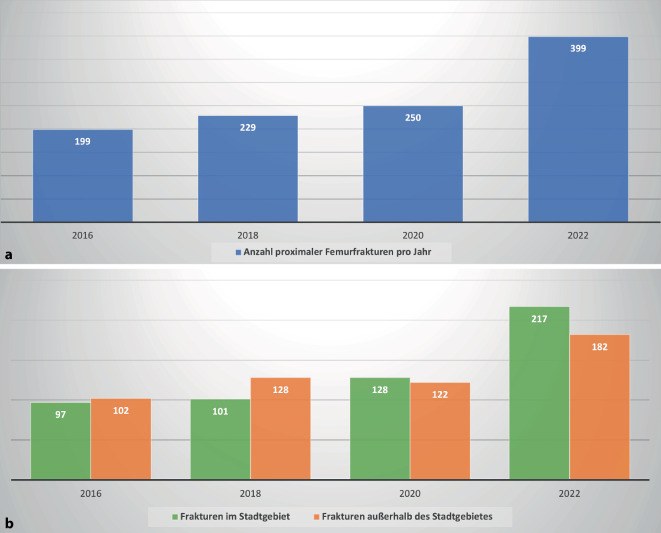

**Figure 2:**
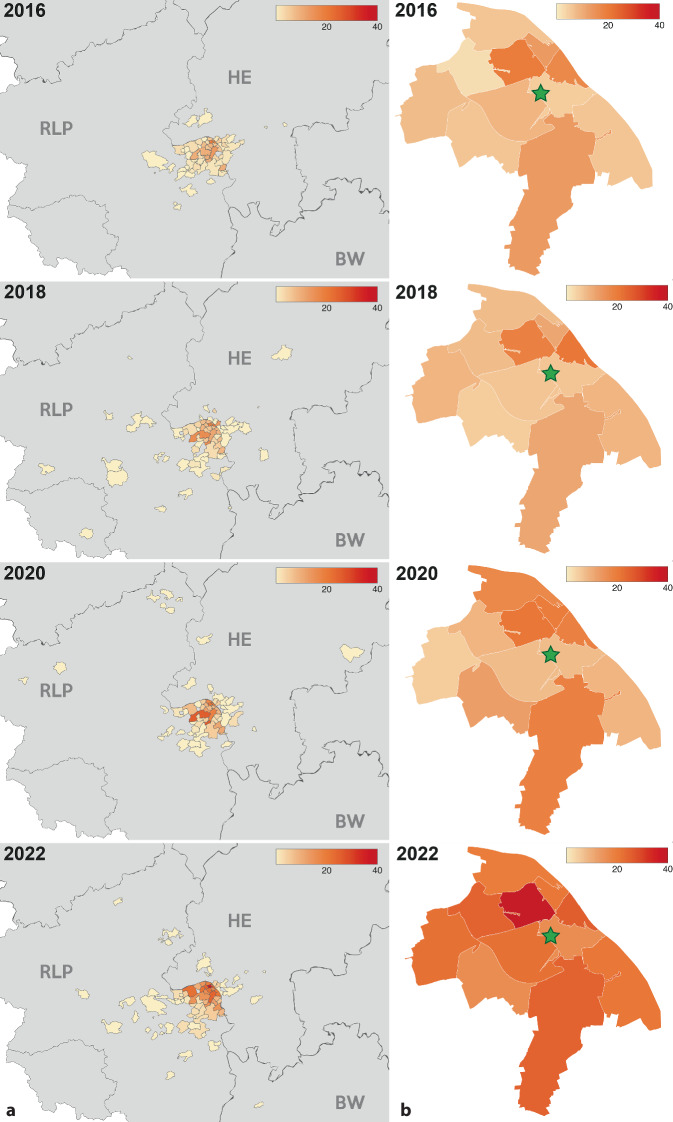

An der untersuchten Klinik wurde in den Jahren 2016–2022 eine steigende Anzahl an proximalen Femurfrakturen versorgt. Im Jahr 2016 waren es 199 Frakturen; im Jahr 2022 waren es 399 (Tab. 1 für jedes einzelne Jahr und Abb. 1 mit Zweijahresintervall zur besseren Übersicht). Der größte Anstieg an versorgten Patienten erfolgte im Zeitraum zwischen 2020 und 2022, mit einer Zunahme um 59,6 %. Insgesamt ist die Zahl der Patienten zwischen 2016 und 2022 um 100 % gestiegen (Abb. 1a). Angestiegen ist ebenfalls die Zahl der versorgten Frakturen aus dem Stadtgebiet der untersuchten Klinik. Bis auf die Veränderung zwischen 2018 und 2020 ist auch die Anzahl an versorgten Frakturen von Patienten mit einem Wohnort außerhalb des Stadtgebietes angestiegen. Im letzten Zeitraum von 2020 bis 2022 betrug dieser Anstieg 49,2 % (Abb. 1b). In Abb. 2 sind dieselben Daten in *Heatmaps* farblich und geografisch veranschaulicht. Es zeigt sich seit 2016 eine Zunahme der Entfernung des Wohnsitzes der versorgten Patienten zur untersuchten Klinik. Weiterhin zeigt sich eine Zunahme der Anzahl der versorgten Patienten im Stadtgebiet (Abb. 2).

**Table 1:** 

Jahr	Operativ versorgte proximale Femurfrakturen (ICD-10 S72.0x und S72.1x)	Veränderung zum Vorjahr
2016	199	–
2017	189	−10 (−0,05 %)
2018	229	+40 (+17,5 %)
2019	217	−12 (−0,06 %)
2020	250	+33 (+13,2 %)
2021	328	+78 (+23,8 %)
2022	399	+71 (+17,8 %)

**Figure 3:**
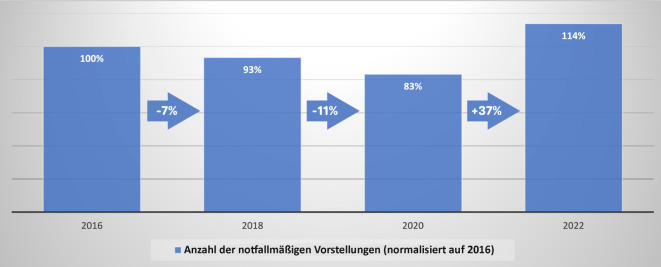

Im Zeitraum von 2016 bis 2022 ist die Anzahl notfallmäßiger Vorstellungen in der Notaufnahme der untersuchten Klinik um 14 % gestiegen (Abb. 3). Die niedrigste Anzahl an Patienten wurde 2020 behandelt. Von 2020 bis 2022 ist die Anzahl der Patienten um 37 % gestiegen.

An der untersuchten Klinik gab es seit 2016 keine strukturellen Veränderungen im Sinne einer Zunahme des ärztlichen und pflegerischen Personals oder der Operationskapazitäten. 2021 ist in dem untersuchten Traumanetzwerk eine Klinik vom regionalen zum lokalen Traumazentrum herabgestuft worden. Eine weitere Klinik in unmittelbarer Nähe (Luftlinie < 10 km) wurde 2020 aufgrund einer Insolvenz geschlossen. Weiterhin ist 2022 eine Klinik als regionales Traumazentrum neu im TraumaNetzwerk hinzugekommen.

## Diskussion

Proximale Femurfrakturen stellen in vielerlei Hinsicht eine Herausforderung in der Versorgung dar. In der ganz überwiegenden Mehrzahl der Fälle ist eine operative Versorgung notwendig, welche durch den G‑BA-Beschluss seit 2019 innerhalb von 24 h zu erfolgen hat. Meist sind geriatrische Patienten betroffen, deren Komorbiditäten und Medikation es zu berücksichtigen gilt. In der vorliegenden Studie wurden die ICD-10-Diagnosen der proximalen Femurfrakturen als Indikator für das notfallmäßige Patientenaufkommen in der unfallchirurgischen Versorgung genutzt. Analysiert wurden die Frakturen, die laut Beschluss des G‑BA von 2019 innerhalb von 24 h versorgt sein müssen. Es ist folglich davon auszugehen, dass fast alle dieser Patienten neben einer Vorstellung in der Notaufnahme auch eine Operation und einen stationären Aufenthalt hatten. Weiterhin sind proximale Femurfrakturen die häufigsten Frakturen Erwachsener in Deutschland. Distale Radiusfrakturen und Sprunggelenkdistorsionen bzw. -frakturen sind zwar häufige Gründe für Vorstellungen in der Notaufnahme, ihre Versorgung ist jedoch deutlich heterogener als die der proximalen Femurfrakturen. So können einige dieser Frakturen konservativ behandelt werden, und die Therapieentscheidung kann zu einem größeren Anteil vom behandelnden Arzt beeinflusst sein. Zudem gibt es hinsichtlich des Operationszeitpunkts keine gesetzlichen Regelungen. Ebenfalls muss nur ein geringerer Anteil dieser Patienten stationär behandelt werden, woraus sich eine veränderte Ressourcennutzung ergibt. Wir entschieden uns somit aufgrund der Homogenität der Behandlung von proximalen Femurfrakturen, diese als guten Indikator für das Patientenaufkommen und den Behandlungsaufwand heranzuziehen.

Da proximale Femurfrakturen innerhalb von 24 h operativ versorgt sein müssen und nie planbar auftreten, müssen sie entweder im Bereitschaftsdienst außerhalb der Kernarbeitszeiten, z. T. mitten in der Nacht, versorgt werden oder sie „verdrängen“ elektiv geplante Operationen aus der Kernarbeitszeit. Eine Vorhaltung von „Notfall-OP“ ist aufgrund der engen Ressourcen kaum noch möglich. Im Jahr 2016 wurden an der untersuchten Klinik 199 proximale Femurfrakturen behandelt. Das bedeutet, dass durchschnittlich an 4 von 7 Tagen eine solche Fraktur operativ versorgt wurde. Im Gegensatz dazu wurden im Jahr 2022 durchschnittlich pro Tag mehr als eine proximale Femurfraktur versorgt. Wurden diese Frakturen im Bereitschaftsdienst (in der Nacht oder am Wochenende) versorgt, ist von einer deutlich höheren Belastung/Inanspruchnahme der Diensthabenden aller beteiligten Professionen im Vergleich zu 2016 auszugehen. Konnten Frakturen nicht im Bereitschaftsdienst versorgt werden, führte dies an Werktagen bei unveränderter OP-Kapazität zu einer Verdrängung elektiver Eingriffe.

In dieser Studie konnte ein signifikanter Anstieg der behandelten Patienten mit proximalen Femurfrakturen zwischen den Jahren 2016 bis 2022 an der untersuchten Klinik festgestellt werden. Hierbei fällt auf, dass der Anstieg v. a. zwischen den Jahren 2020 und 2022 besonders groß war. Im Jahr 2020, das v. a. durch die COVID-19-Pandemie und längere „Lockdowns“ gekennzeichnet war, wurden nicht weniger, sondern mehr proximale Femurfrakturen als in den Vorjahren behandelt. Dies steht im Gegensatz zu einem in der Literatur beschriebenen Rückgang der Inzidenzen proximaler Femurfrakturen auf nationaler und internationaler Ebene während des Beginns der COVID-19-Pandemie 2020 [2, 7, 10] und auch im Gegensatz zu einem Abfall der versorgten Patienten in der Notaufnahme der untersuchten Klinik im Jahr 2020 (Abb. 3). Insgesamt ist die Anzahl der notfallmäßigen Vorstellungen an der untersuchten Klinik von 2016 bis 2022 um 14 % gestiegen. In Deutschland ist die Inzidenz der proximalen Femurfrakturen von 2009 bis 2019 um ca. 24 % gestiegen [8]. Der hierzu im Vergleich überproportionale Anstieg an operativ versorgten Patienten an der untersuchten Klinik (100 % in 6 Jahren) kann folglich nur dadurch erklärt werden, dass sich andere Krankenhäuser in geringerem Ausmaß an der Behandlung beteiligten und die Patienten deshalb öfter an einen ÜTZ transferiert werden mussten. Eine weitere Erklärungsmöglichkeit hierfür ist das durch den G‑BA vorgegebene Zeitfenster der operativen Versorgung. Zuvor war das Zeitfenster von 24 h zwar seit längerer Zeit Gegenstand der Empfehlungen [1, 11], seit dem Inkrafttreten des Beschlusses des G‑BA muss eine Klinik jedoch bei Nichteinhaltung mit Konsequenzen und monetären Sanktionen rechnen. Diese reichen von aufwendigen Stellungnahmen über Wegfall des Vergütungsanspruches bis zum Entzug der Versorgungsgenehmigung. Dies kann gerade für kleinere Häuser abschreckend sein und einen Anreiz schaffen, sich nicht an der Versorgung zu beteiligen. Da dieser Beschluss Ende 2019 in Kraft trat, kann er auch den starken Anstieg der versorgten Frakturen im Zeitraum von 2020 bis 2022 erklären.

Bei Analyse der Heatmaps (Abb. 2) fällt bereits vor der COVID-19-Pandemie eine Zunahme der Anzahl an Patienten mit weiter entferntem Wohnort auf, wobei viele Patienten aus benachbarten TraumaNetzwerken stammen. Weiterhin wurden bereits vor der COVID-19-Pandemie mehr Patienten aus dem Stadtgebiet an der untersuchten Klinik versorgt. Dieser Prozess wurde seit der COVID-19-Pandemie noch einmal deutlich verstärkt. Dies lässt die Vermutung zu, dass die COVID-19-Pandemie zu nachhaltigen Versorgungsengpässen mit Betten‑, Personal- und Ressourcenmangel an kleineren Krankenhäusern geführt hat. Folglich können diese sich nicht mehr ausreichend an der unfallchirurgischen Notfallversorgung beteiligen, und die Patienten müssen weitere Strecken bis zum versorgenden Krankenhaus zurücklegen. Gerade für ältere Patienten ist jedoch eine gewisse Nähe zu den Angehörigen im Sinne einer Delirprophylaxe vorteilhaft [9]. Sollte sich dieser Trend auch in anderen Frakturentitäten bzw. anderen medizinischen Disziplinen bestätigen, ist bereits jetzt von einer inhomogenen Notfallversorgung in Deutschland auszugehen, die einer Ressourcenanpassung der Kapazitäten bedarf. Insbesondere aber sind Regelungen und Finanzierung von Vorhaltungen und Vorhaltungsmaßnahmen wie zusätzliche Notfall-OP mit entsprechendem Personalschlüssel notwendig, um einem Kollaps der nichtangepassten Notfallressourcen entgegenzuwirken.

Insgesamt konnte in dieser Studie ein im Vergleich zur Steigerung der nationalen Inzidenz überproportionaler Anstieg der versorgten proximalen Femurfrakturen an einem ÜTZ gezeigt werden. Hierdurch ist von einem deutlichen Anstieg der Belastung für alle beteiligten Professionen auszugehen. Studien prognostizieren eine Zunahme der Gesamtzahl aller Frakturen um ca. 35 % von 2019 bis 2025 [8]. Bei einer immer älter werdenden Gesellschaft [12] ist jedoch von einem überproportionalen Anstieg der proximalen Femurfrakturen in den kommenden Jahren auszugehen. Es bleibt zu klären, warum viele Patienten eine so weite Strecke bis zur versorgenden Klinik zurücklegen mussten. Sollte dies mit den Vorgaben des G‑BA zusammenhängen, hätte diese Maßnahme, die eigentlich eine bessere Versorgung der Patienten garantieren sollte, einen gegenteiligen Effekt. Weitere Studien sind notwendig, um die Situation in anderen TraumaNetzwerken und die Gründe für die Veränderung der Versorgungsstruktur zu eruieren, um dies in Umstrukturierungsmaßnahmen einfließen zu lassen.

### Limitationen

Um die Aussagekraft der Untersuchungsergebnisse zu erhöhen, wären u. a. folgende Änderungen notwendig:

- Betrachtung anderer Kliniken und ÜTZ,
- Analyse und Vergleich anderer Frakturentitäten.

## Fazit für die Praxis

Multizentrische Studien zur aktuellen Analyse der Versorgungsrealität sind notwendig. Weiterhin ist eine Optimierung des bundeslandübergreifenden Informationsaustausches verschiedener Systeme erforderlich; erste Ansätze existieren in wenigen Regionen. Außerdem sollte idealerweise ein einheitliches und flächendeckend verfügbares Informationssystem für den interdisziplinären Versorgungsnachweis in der ganzen Bundesrepublik etabliert werden. Zuletzt sollten Zwänge und Grenzen des G‑BA-Beschlusses 4069 im Hinblick auf die Versorgungsrealität in der aktuellen Krankenhauslandschaft kritisch bewertet und diskutiert werden.
